# Case Report: *KMT2A* amplification in two adult patients with B-cell acute lymphoblastic leukemia

**DOI:** 10.3389/fonc.2026.1656404

**Published:** 2026-02-26

**Authors:** Min Gao, Yunjia Chen, Kimo Bachiashvili, Pankit J. Vachhani, Omer Jamy, Shuko Harada, Alexander Craig Mackinnon, Nirupama Singh, Aishwarya Ravindran, Baleed Vishnu Reddy, Andrew J. Carroll, Fady M. Mikhail

**Affiliations:** 1 Department of Genetics, University of Alabama at Birmingham, Birmingham, AL, United States; 2 Department of Medicine (Hematology/Oncology), University of Alabama at Birmingham, Birmingham, AL, United States; 3 Department of Pathology, University of Alabama at Birmingham, Birmingham, AL, United States

**Keywords:** B-ALL, complex karyotype, *CRLF2* rearrangement, *KMT2A* amplification, *TP53* variant

## Abstract

The *KMT2A* gene, located at chromosome band 11q23, encodes a lysine methyltransferase essential for hematopoietic gene regulation. While *KMT2A* rearrangements are common in acute myeloid leukemia (AML) and B-cell acute lymphoblastic leukemia (B-ALL), *KMT2A* amplification is rare, occurring in ~1% of AML cases and even less frequently in B-ALL. Given its rarity, understanding *KMT2A* amplification in B-ALL is crucial for improving diagnostics and therapy. We report two adult B-ALL cases with *KMT2A* amplification. Patient 1, a 58-year-old male, had *KMT2A* amplification (6~18 copies in 68.5% of bone marrow cells), a complex karyotype, and a pathogenic *TP53* variant (c.524G>A, p.Arg175His). He underwent induction chemotherapy but passed away after two months due to complications. Patient 2, a 66-year-old female, had *KMT2A* amplification (8~11 copies in 87.5% of peripheral blood cells) and *CRLF2* rearrangement, representing the first reported case of *de novo* Ph-like B-ALL with *KMT2A* amplification in an adult. She deteriorated rapidly and died within four days. In addition to these two cases from our cohort, we review nine published cases with *KMT2A* amplification in B-ALL, which showed frequent *TP53* alterations, emphasizing the clinical and genetic characteristics of this aggressive leukemia subtype. These cases highlight the high-risk nature of *KMT2A*-amplified B-ALL, particularly in older adults, where prognosis is poor and linked to *TP53* variants or *CRLF2* rearrangement. Our review underscores the need for genetic profiling to improve risk stratification and treatment. Given the limited documented cases, further research is essential to develop better therapeutic strategies for *KMT2A*-amplified B-ALL.

## Introduction

*KMT2A* (lysine methyltransferase 2A), formerly known as *MLL* (mixed-lineage leukemia), is a gene on chromosome band 11q23, which encodes a histone methyltransferase that methylates H3K4, activating key developmental genes and regulating self-renewal and differentiation in hematopoietic stem cells (1). *KMT2A* rearrangements are frequently observed in both myeloid and lymphoid leukemias, particularly in acute myeloid leukemia (AML) and B-cell acute lymphoblastic leukemia (B-ALL) (2, 3). Unlike *KMT2A* rearrangements, which involve the fusion of *KMT2A* with various partner genes, *KMT2A* amplification arises through mechanisms such as extrachromosomal double minutes or intrachromosomal tandem duplications, often presenting as homogeneously staining regions (HSRs) (4). *KMT2A* amplification is a rare genetic abnormality, occurring in approximately 1% of AML cases (5–7) and even less frequently in B-ALL, with only a few reported cases in the literature (8, 9).

Despite its rarity in B-ALL, *KMT2A* amplification has been associated with distinct clinical and genetic features. One notable aspect is its frequent association with *TP53* pathogenic variants, including single nucleotide variants or structural variants, further contributing to the aggressive nature of the disease (9). *TP53* is a crucial tumor suppressor that regulates genomic stability and apoptosis. *TP53* pathogenic variants impair its function, leading to uncontrolled proliferation, therapy resistance, and poor clinical outcomes (10, 11). *KMT2A*-amplified B-ALL cases with *TP53* pathogenic variants are particularly challenging to treat, demonstrating limited responses to standard therapies and a high risk of relapse (9). Given this association, understanding the interplay between *KMT2A* amplification and *TP53* variants is crucial for risk stratification and development of targeted therapies to improve patient outcomes.

*CRLF2* rearrangements, a hallmark of Ph-like B-ALL, contribute to cytokine receptor-mediated leukemogenesis by activating the JAK-STAT pathway, leading to enhanced proliferation and poor prognosis (12, 13). About 55–68% of *CRLF2*-rearranged cases also have JAK-pathway mutations (most often *JAK2*) (14). *CRLF2* rearrangements have not been documented in *KMT2A*-amplified B-ALL. Recent adult cohort data from a resource-limited setting confirmed that Ph-like B-ALL is common and enriched for adverse features such as MRD positivity and *IKZF1* deletions, underscoring its aggressive clinical behavior (15). Since both *CRLF2* rearrangements and *KMT2A* amplification define high-risk B-ALL subtypes and are associated with treatment resistance and poor clinical outcomes, it remains unclear whether they coexist or represent distinct oncogenic mechanisms. If both alterations co-occur, epigenetic dysregulation and JAK-STAT hyperactivation may drive an ultra-high-risk leukemia, requiring combined JAK inhibitors and epigenetic modulators. Further research is needed to assess their coexistence, interactions, and treatment strategies.

Given the rarity of *KMT2A* amplification in B-ALL, systematic documentation and analysis of cases are essential to improve our understanding of its pathogenesis and refine the diagnostic and therapeutic strategies. In this study, we report two adult B-ALL patients with complex karyotypic abnormalities and *KMT2A* amplification, including one patient with a *TP53* pathogenic variant (p.Arg175His) and another with a *CRLF2* rearrangement, marking the first documented case of *KMT2A* amplification coexisting with Ph-like B-ALL in an adult. Additionally, we review our cases along with nine recently reported cases, providing further insight into the clinical, cytogenetic, and molecular characteristics of *KMT2A*-amplified B-ALL, and addressing associated management challenges.

## Materials and methods

### Flow cytometry analysis

Bone marrow specimens from our patients underwent flow cytometry analysis at the University of Alabama at Birmingham (UAB) Pathology Lab. The analysis was conducted following standard flow cytometry protocols to ensure accuracy and reproducibility. The cell markers analyzed by flow cytometry included T-cell markers (CD2, CD3, CD4, CD5, CD7, CD8), B-cell markers (CD9, CD10, CD19, CD20, CD22, Kappa, Lambda), myeloid and progenitor markers (CD13, CD14, CD15, CD16, CD33, CD34, CD38, CD58, CD64, CD117), and the pan-leukocyte marker CD45.

### G-banded chromosome and FISH analyses

Bone marrow or peripheral blood specimens from our patients underwent comprehensive cytogenomic analyses, including G-banded chromosome and fluorescence *in situ* hybridization (FISH) analyses, at the UAB Cytogenetics Lab. G-banded chromosome analysis was performed using standard cytogenetic protocols. Interphase and/or metaphase FISH analyses were conducted following the manufacturer’s protocols, as previously described (16). The B-ALL FISH panel (Abbott) included *BCR*/*ABL1* dual-fusion probes, *KMT2A* break-apart probes, *ETV6*/*RUNX1* dual-fusion probes, and centromeric probes for chromosome 4, 10, and 17. The Ph-like B-ALL FISH panel (OGT Cytocell) included break-apart probes for *ABL2* (1q25.2), *PDGFRB* (5q32), *JAK2* (9p24.1), *ABL1* (9q34.1), and *CRLF2* (Xp22.33/Yp11.32). The *TP53* probe mixture (Abbott) includes a *TP53* (17p13.1) probe and a chromosome 17 centromere probe. Findings were reported in accordance with the International System for Human Cytogenomic Nomenclature (ISCN) 2020.

### Targeted next-generation sequencing panel analysis

Targeted next-generation sequencing (NGS) panel analysis was conducted on bone marrow biopsy samples at the UAB Pathology Lab to detect gene fusions and somatic variants using an RNA-based myeloid fusion panel and a myeloid mutation panel, following established protocols (17, 18). The RNA-based myeloid fusion panel targeted key fusion driver genes, including *ABL1, ABL2, BCL2, BRAF, CCND1, CREBBP, EGFR, ETV6, FGFR1, FGFR2, FUS, HMGA2, JAK2, KAT6A (MOZ), KAT6B, KMT2A, KMT2A-PTDs, MECOM, MET, MLLT10, MRTFA (MKL1), MYBL1, MYH11, NTRK2, NTRK3, NUP214, NUP98, PAX5, PDGFRA, PDGFRB, RARA, RUNX1, TCF3, TFE3*, and *ZNF384*. The myeloid mutation panel assessed hotspot variants in genes such as *ABL1, ANKRD26, BRAF, CBL, CSF3R, DDX41, DNMT3A, FLT3, GATA2, HRAS, IDH1, IDH2, JAK2, KIT, KRAS, WT1, MPL, MYD88, NPM1, NRAS, PPM1D, PTPN11, SETBP1, SF3B1, SMC1A, SMC3, SRSF2*, and *U2AF1*, along with full-gene sequencing of *ASXL1, BCOR, CALR, CEBPA, ETV6, EZH2, IKZF1, NF1, PHF6, PRPF8, RB1, RUNX1, SH2B3, STAG2, TET2, TP53*, and *ZRSR2*.

### Data visualization and statistical analysis

Data visualization and statistical analysis were performed in R (v.4.3.1). A violin plot was generated using the ggviolin function in the ggpubr (v.0.6.0) package in R, while pie charts were created using the pie3D function in the plotrix (v.3.8-6) package. These visualizations illustrate the age distribution, clinical outcomes, and sex distribution of 11 B-ALL patients with *KMT2A* amplification, with the violin plot representing age distribution and the pie chart depicting clinical outcomes and sex proportions. Tables summarizing patient characteristics and analytical results were generated using the GT (v.0.10.1) package, ensuring clear and structured data presentation. A Student’s t-test was applied for comparisons between deceased and remission groups, with a p-value < 0.05 considered statistically significant.

## Case presentations

### Patient 1

**Figure 1:**
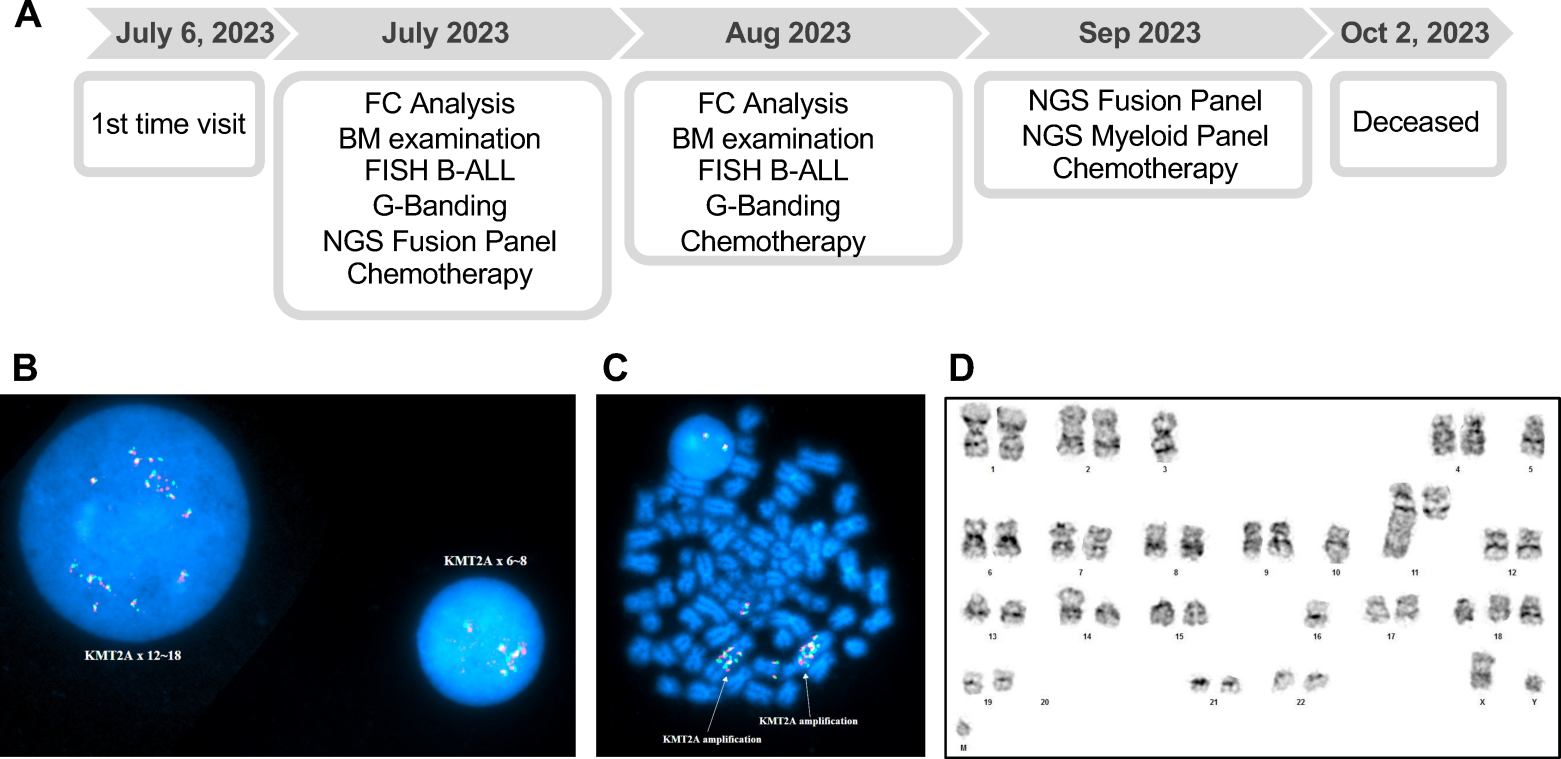
**(A)** Timeline of diagnostic evaluations and clinical course of Patient 1. FC: Flow Cytometry, BM: Bone Marrow, FISH: Fluorescence *in situ* hybridization, B-ALL: B-cell acute lymphoblastic leukemia panel. **(B)** Interphase and **(C)** metaphase FISH analyses of Patient 1 unstimulated bone marrow (BM) cells using the *KMT2A* BAP, showing *KMT2A* amplification (6~18 copies). **(D)** G-banded chromosome analysis of Patient 1 unstimulated BM cells, demonstrating a stemline clone with complex karyotype involving chromosomes 3, 5, 10, 11, 14, 16, 18, and 20, in addition to the presence of 1~3 marker chromosomes.

A 58-year-old male with a history of hypertension (HTN), hyperlipidemia (HLD), gastroesophageal reflux disease (GERD), and anxiety/depression was transferred from an outside hospital for suspected newly diagnosed acute leukemia. He was admitted to UAB for comprehensive diagnostic evaluation. The patient’s clinical course and diagnostic workup are shown in **Figure 1A**. Flow cytometry of the bone marrow aspirate identified 54.11% leukemic blasts, positive for CD13dim, CD19, CD22dim, CD34, CD38, CD45, CD58, and nTdT, and negative for NK/T, other myeloid, and monocytic markers, confirming a B-ALL diagnosis (**Supplementary Table S1**). Bone marrow morphology assessment revealed a hypercellular marrow (approximately 90% cellularity) with markedly reduced trilineage hematopoiesis. Most blasts on touch imprint exhibited cytoplasmic vacuolization. These findings are consistent with B-ALL.

Interphase FISH analysis of uncultured bone marrow cells, using the B-ALL FISH panel, identified 6~18 copies of *KMT2A* in 68.5% of cells (**Figures 1B, C**), along with copy number alterations affecting several genes, including *ABL1* (3~5 copies in 68% of cells), *ETV6* (2~5 copies in 67.5% of cells), *RUNX1* (3~4 copies in 9.5% of cells), and chromosomes 4, 10, and 17 (3~5 copies each) (**Supplementary Table S1**). G-banded chromosome analysis of unstimulated bone marrow cells revealed the presence of a stemline clone with complex karyotype involving chromosomes 3, 5, 10, 11, 14, 16, 18, and 20, in addition to the presence of 1~3 marker chromosomes, and a subclone that represents the inexact doubling product of the stemline clone: 41~44,XY,-3,-5,-10,add(11)(q23),add(14)(p11.2),-16,add(18)(q23),-20,+1~3mar[cp4]/70~80<3n>,XXY,+2,-3,-5,+6,-10,+11,add(11)(q23)x2,+13,+13,add(14)(p11.2),+15,-16,add(16)(q24),add(18)(q23),+21,+22,+2~5mar[cp10] (**Figure 1D**).

A targeted myeloid NGS panel identified a clinically significant *TP53* missense variant (c.524G>A, p.Arg175His) with a 91% variant allele frequency (VAF) (**Supplementary Table S1**), suggesting a potential role in leukemogenesis and treatment resistance. Additionally, no fusion transcripts were detected using a targeted RNA-based myeloid fusion NGS panel.

The patient was started on induction chemotherapy (**Figure 1A**), which was complicated by febrile neutropenia, necessitating the removal of his mediport as a potential infection source. During hospitalization, he developed progressive hypoxia, pleural effusions, and encephalopathy, requiring multiple Medical Emergency Team (MET) activations, including one for unresponsiveness, which improved with Narcan. Bronchoscopy showed minimal secretions and arytenoid inflammation, suggesting aspiration. His condition worsened, requiring MET activation for altered mental status and hypotension. He developed severe lactic acidosis (lactate level of 11 mmol/L), tested positive for COVID19, and had persistent pseudomonas and klebsiella bacteremia. Despite maximum vasopressor support, he remained profoundly hypotensive. He ultimately succumbed to refractory septic shock and multiorgan failure after a two-month hospitalization.

### Patient 2

**Figure 2:**
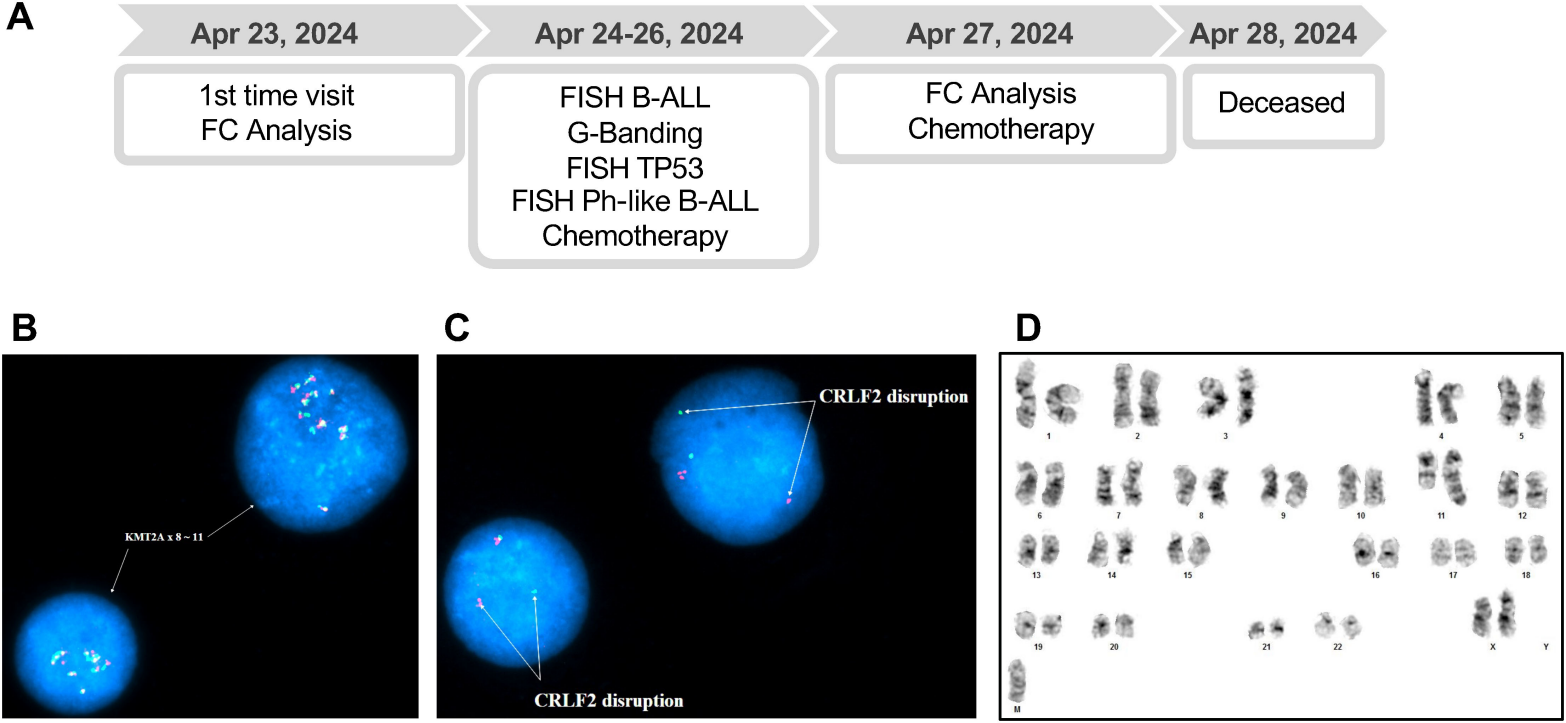
**(A)** Timeline of diagnostic evaluations and clinical course of Patient 2. FC: Flow Cytometry, FISH: Fluorescence *in situ* hybridization, B-ALL: B-cell acute lymphoblastic leukemia panel. **(B)** Interphase FISH analysis of Patient 2 peripheral blood cells using the *KMT2A* BAP, showing *KMT2A* amplification (8~11 copies). **(C)** Interphase FISH analysis of Patient 2 unstimulated BM cells using the *CRLF2* BAP, demonstrating *CRLF2* rearrangement. **(D)** G-banded chromosome analysis of Patient 2 peripheral blood cells were unsuccessful; however, limited analysis revealed additional material of unknown origin attached to 11q23.

A 66-year-old female with a history of type 2 diabetes mellitus, GERD, HTN, obstructive sleep apnea, and HLD presented to an outside hospital with dizziness, ear drainage, gait difficulty, shortness of breath, and cough. Patient’s workup revealed leukocytosis, anemia, and thrombocytopenia, raising suspicion for acute leukemia. She was transferred to UAB for further evaluation. The patient’s diagnostic timeline and treatment course are illustrated in **Figure 2A**. Flow cytometry of the peripheral blood demonstrated 89.71% leukemic blasts that were positive for CD10, CD13, CD19, CD22dim, CD34, CD38, CD58, nTdT, and negative for NK/T, other myeloid, and monocytic cell markers, supporting a diagnosis of B-ALL (**Supplementary Table S1**).

Interphase FISH analysis using the B-ALL FISH panel identified *KMT2A* amplification, with 8~11 copies detected in 87.5% of peripheral blood cells (**Figure 2B**). Additionally, the Ph-like B-ALL FISH panel analysis detected a *CRLF2* gene rearrangement in 85% of bone marrow cells (**Figure 2C**), with the classic fusion signal disruption suggestive of the t(X;14)(p22.3;q32.3) and resulting in *IGH::CRLF2* fusion. This is an alteration commonly associated with high-risk leukemia and poor treatment response. Further FISH analysis of the *TP53* gene in bone marrow cells showed no deletions or structural rearrangements (**Supplementary Table S1**). G-banded chromosome analysis of peripheral blood cells was unsuccessful, but limited analysis indicated additional material attached to chromosome 11 at band 11q23 (**Figure 2D**), a region frequently involved in *KMT2A*-related leukemias.

During hospitalization, the patient developed pulmonary edema and decompensated cirrhosis, accompanied by altered mental status and respiratory distress, requiring MET activation, Bilevel Positive Airway Pressure support, and eventual intubation. Despite treatment with Lasix, Solumedrol, broad-spectrum antibiotics, and blood transfusions, her condition worsened due to septic shock, acute kidney injury requiring continuous renal replacement therapy, non-ST elevation myocardial infarction, and cirrhosis with varices. Given her multiorgan failure and poor prognosis, goals of care discussions led to a comfort care transition. She was later found unresponsive with no pulse, and electrocardiogram confirmed no cardiac rhythm. She passed away four days after hospitalization (**Figure 2A**). Due to her rapid clinical deterioration and subsequent death, targeted NGS could not be performed.

### Cases review

**Figure 3:**
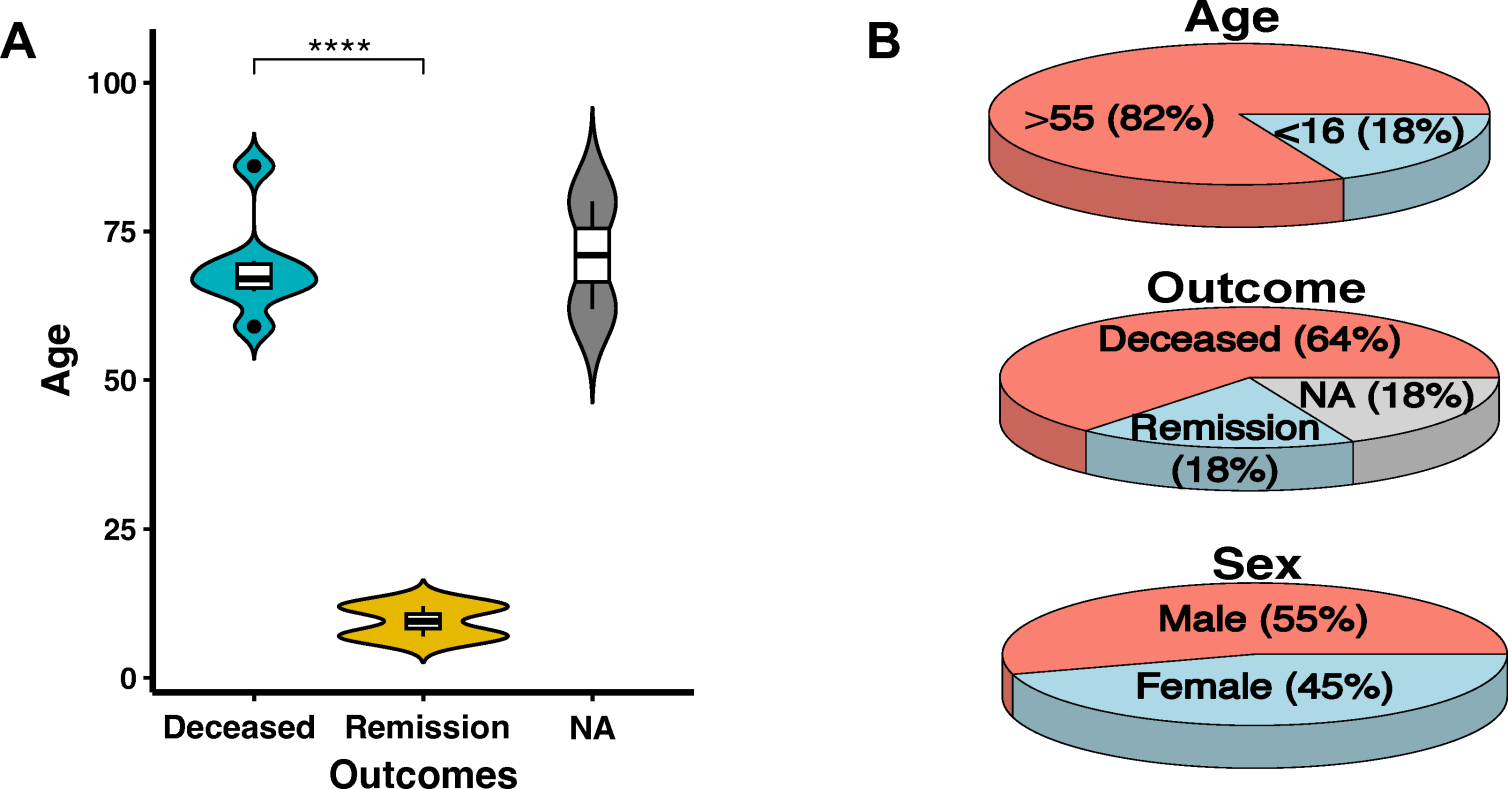
**(A)** Violin plot showing age and outcome distribution of B-ALL patients with *KMT2A* amplification (our two patients and nine published cases). Outcomes: Deceased (blue), Remission (yellow), NA (not available) (gray). ****p-value < 0.001. **(B)** Pie charts summarizing age (82% >55 years, 18% <16 years), outcome (64% deceased, 18% remission, 18% NA), and sex (55% male, 45% female) distribution.

This report reviews two cases from our cohort and nine published cases of *KMT2A*-amplified B-ALL (**Table 1**), summarizing distributions by age (9/11, 82% >55 years; 2/11, 18% <16 years), outcome (7/11, 64% deceased; 2/11, 18% in remission; 2/11, 18% not available), and sex (6/11, 55% male; 5/11, 45% female). Both pediatric patients (6, 19) (18%) achieved remission, whereas the majority of adult patients (64%) experienced fatal outcomes, suggesting a strong age-associated difference in clinical course. Patients in the remission group clustered at markedly younger ages than those in the deceased group, and fatal cases were predominantly observed among older adults (**Figures 3A, B**). Among the eleven cases analyzed, one case (9%) was classified as Ph-like B-ALL, two cases (18%) as pre-B ALL (9, 20), three cases (27%) as therapy-related B-ALL (21, 22), and one case (9%) exhibited transformation from B-ALL to T-ALL (23). CD10 expression varied, with three cases positive (27%) and seven negative (63%), indicating immunophenotypic diversity. *KMT2A* amplification (3 to >20 copies) was frequently associated with complex karyotypes, particularly involving chromosome band 11q23. *TP53* alterations were found in five cases (45%), including missense variants or structural variants. Additional genetic changes included *CRLF2* rearrangement (1/11, 9%), multiple copies of *ABL1, BCR, ETV6*, and *RUNX1*, as well as chromosomal deletions. These findings highlight the need for further research and novel therapies for this high-risk leukemia subtype.

**Table 1:** Clinical features of B-ALL patients with *KMT2A* amplification.

Case	Reference	Sex	Age (Yrs)	Diagnosis	CD10 (FC)	Karyotype	*KMT2A* (FISH)	*TP53*	Other results	Outcome
1	Current case 1	M	59	B-ALL	Neg	41~44,XY,-3,-5,-10,add(11)(q23),add(14)(p11.2),-16,add(18)(q23),-20,+1~3mar[cp4]/70~80<3n>,XXY,+2,-3,-5,+6,-10,+11,add(11)(q23)x2,+13,+13,add(14)(p11.2),+15,-16,add(16)(q24),add(18)(q23),+21,+22,+2~5mar[cp10]	6~18	c.524G>A, p.Arg175His, VAF 91%	3–5 copies of *ABL1, BCR, ETV6, RUNX1*, chr 4, 10, 17.	Deceased
2	Current case 2	F	66	Ph-like B-ALL	Pos	Chromosome analysis failed, but metaphases revealed unknown material on chromosome 11q23	8~11	No deletion/duplication	Pos for *CRLF2*-R	Deceased
3	PMID: 12604431	F	86	pre-B ALL	Neg	44,XX,del(5)(q13q31),dic(6;17)(p25;q11),hsr(11)(pter_q23::hsr::q21::hsr::qter),-16,-17[18]/46,XX[2]	Multiple	Chr 17 loss	NA	Deceased
4	PMID: 23238285_1	M	80	Therapy-related B-ALL	Neg	42,XY,−3,hsr(11)(q23),−14,−16,−20[cp6]/43,idem,+mar[cp3]/46,XY[11]	Multiple	NA	Neg for *BCR::ABL1, FLT3*	NA
5	PMID: 38735761	F	70	pre-B ALL	Neg	44-47,XX,del(5)(q22q35),-7,dic(12;18)?(?p12;p11.3),der(15)t(?7;15)(p11.2;p11.2),-20, del(22)(q13.1),+1~4mar[cp6]/44,idem,der(11),add(11)(p15)add(11)(q23)[cp6]/83–87<4n>,XXXX,-2,-3,-4,del(5)(q22q35)x2,-7,-9,-11,add(11)(q?23)x2,-13,-14,-15,-15,-19,-20,-21,+8~10mar[cp4]/46,XX[2]	≥4	Biallelic p.Val173Met, p.Val216Met, VAFs 37% and 40%, respectively	NA	Deceased
6	PMID: 36964033	M	69	B-ALL	Neg	NA	>20	Deletion	3–4 copies of *BCR, ABL1*	Deceased
7	Proc UCLA Healthc 19 (2015)	F	67	Therapy-related B-ALL	Pos	45-46,XX,add(8)(q24.3),add(16)(q22),+21,del(21)(q22),-22,add(22)(q11.2),+mar[cp8]/46,XX[8]	3~4	NA	Neg for *BCR::ABL1*	Deceased
8	PMID: 35402256	F	65	B-ALL to T-ALL	Neg	44,X,-X,add(1)(p13),add(2)(q21),-4,-5,-10,del(11)(q)?,-12,-14,-17,-18,+r1,+mar1, +mar2,+mar3,+mar4,+mar5 [3]/46,XX[4]	8	c.455dupC, p.Pro153Alafs*28, VAFs 49.5-94.1%	Neg for *BCR::ABL1*	Deceased
9	PMID: 23238285_2	M	62	Therapy-related B-ALL	Neg	44-45,XY,−5,hsr(11)(q23),−15,+mar1[cp3]/48–50,sl,+6,+8,+20,+22[cp2]/57−59,sdl1,+1,+del(1)(q12),+2,+7,+10,+hsr(11)(q23)a,+12,+13,+21,−mar1,+mar2(cp3)/59,sdl2,+X,−1,−del(1)(q12),+del(1)(q25),+5,+10,+11[cp6]/73–77,sdl2,+Y,+del(1)(q12),+3,+4,+5,+6,+8,+9,+10,+11,−hsr(11),+12,+13,+14,+15,+16,+17,+17,+18,+18,+19,+22,−mar2,+mar3x2[cp7]	Multiple	NA	Neg for *FLT3*	NA
10	PMID: 22052166	M	12	B-ALL	Pos	47,X,-Y,+1,del(1)(q25),del(1)(q12),dup(11)(q24q23),add(12)(p11.2),+21	3	NA	NA	Remission
11	PMID: 11069023 (case 7)	M	7	B-ALL	NA	Failed	>2	NA	Failed for SB	Remission

VAF, Variant Allele Frequency; B-ALL, B-cell Acute Lymphoblastic Leukemia; *BCR::ABL1*, *BCR::ABL1* Fusion; Chr, Chromosome; *CRLF2*-R, *CRLF2* Rearrangement; FC, Flow Cytometry; F, Female; FISH, Fluorescence in Situ Hybridization; *FLT3*, *FLT3* Variant; *KMT2A*, *KMT2A* Copies; M, Male; NA, Not Available; Ph-like, Philadelphia Chromosome-Like; SB, Southern Blot; Yrs, Years; Pos, Positive; Neg, Negative.

## Discussion

*KMT2A* amplification is a rare genetic abnormality in B-ALL, with few documented cases (**Table 1**). The aggregated cases summarized in **Table 1** indicate that *KMT2A*-amplified B-ALL predominantly affects older adults, is frequently associated with complex karyotypes involving chromosome band 11q23, a high rate of *TP53*-related alterations including *TP53* variants in three cases and loss of chromosome 17 in one case (45%), CD10 negativity (63%), and carries a poor overall prognosis, with nearly two-thirds of reported patients deceased. Our findings further confirm its strong association with *TP53* pathogenic variants. Patient 1 exhibited a clinically significant *TP53* missense variant (c.524G>A, p.Arg175His, VAF 91%), a known driver of genomic instability and poor prognosis in hematologic malignancies. This variant disrupts DNA binding, impairing tumor suppression, and promoting genomic instability, therapy resistance, and cancer progression. Additionally, this variant exhibits oncogenic properties, enhancing proliferation, invasion, metabolic reprogramming, and angiogenesis (24). *TP53* single nucleotide variants or structural variants were also identified in five previously reported cases, supporting its role as a cooperative oncogenic event in *KMT2A*-amplified B-ALL. Case 3 exhibited a chromosome 17 loss (20), while case 5 had biallelic *TP53* variants (p.Val173Met and p.Val216Met, VAFs 37% and 40%, respectively) (9), suggesting significant *TP53* loss. Case 6 showed a *TP53* deletion (8), reinforcing the link between *TP53* loss and genomic instability, while case 8 carried a *TP53* frameshift variant (c.455dupC, p.Pro153Alafs*28, VAFs 49.5–94.1%) (23). Given the frequent co-occurrence of *TP53* pathogenic variants and *KMT2A* amplification, future studies should explore *TP53*-targeted therapies for this high-risk B-ALL subset. Prior work in therapy-related leukemia has reported an association between *KMT2A* copy-number gain and germline *TP53* alterations (25). However, germline testing was not performed for our patients due to lack of an appropriate non-tumor specimen, and family history was non-contributory based on available documentation. Notably, a very high variant allele fraction (VAF) of 91% was observed for the *TP53* variant in Patient 1, which may reflect a homozygous *TP53* variant due to copy-neutral loss of heterozygosity (LOH) or a hemizygous *TP53* variant resulting from a deletion of the other allele involving this region. However, in the absence of germline testing or detailed family history, it is uncertain what underlies the high allele frequency for this *TP53* variant and whether this variant is germline. Future studies should systematically evaluate germline *TP53* status in this rare subgroup to clarify inherited versus acquired risk.

*KMT2A* abnormalities in B-ALL are most described as *KMT2A* rearrangements, which are frequent in infant ALL. In contrast, *KMT2A* amplification is a mechanistically distinct alteration involving copy-number gain rather than gene fusion. Although *KMT2A* rearrangements have been reported in Ph-like ALL, *KMT2A* amplification has not previously been described in adult Ph-like B-ALL, to the best of our knowledge. Increased *KMT2A* dosage may lead to partial functional convergence with rearranged cases through dysregulation of shared transcriptional programs such as the *HOXA/MEIS1* axis. In this context, this case report identifies the first documented co-occurrence of *KMT2A* amplification and *CRLF2* rearrangement in an adult with *de novo* Ph-like B-ALL. However, targeted NGS could not be performed for Patient 2 due to rapid clinical deterioration, representing a limitation of this study and an important area for future investigation. *CRLF2* rearrangements, a hallmark of Ph-like B-ALL, result in either *IGH::CRLF2* or *P2RY8::CRLF2* fusion with *CRLF2* overexpression, which drive JAK-STAT activation, and are associated with poor treatment response and high relapse rates (12, 13). While well-studied in Ph-like B-ALL, their co-presence in *KMT2A*-amplified cases remains unreported. Although clonal co-localization could not be directly demonstrated, the high percentage of both abnormalities and the presence of a single dominant blast population strongly support their occurrence within the same leukemic clone, suggesting particularly aggressive disease biology.

Recognition of *KMT2A* amplification, especially with *TP53* alterations and/or *CRLF2* rearrangement, has management implications beyond prognosis and should prompt early, genomically informed escalation of care. Menin inhibitors (revumenib, ziftomenib) represent a rational strategy to disrupt *KMT2A*-associated transcriptional programs (26, 27), while JAK inhibition (ruxolitinib) is supported for *CRLF2*-rearranged Ph-like B-ALL (28). Combined pathway targeting may be considered in dual-positive cases. Given poor outcomes with conventional chemotherapy, early clinical trial referral is warranted, and immunotherapies (blinatumomab, inotuzumab, CAR-T) may serve as bridge or salvage options (29–31).

Cytogenetic complexity is another key feature of *KMT2A*-amplified B-ALL. Both patients exhibited complex karyotypes with multiple chromosomal abnormalities, particularly involving chromosome band 11q23, consistent with previous reports linking *KMT2A* amplification to genomic instability. Additional copy number abnormalities in genes such as *ABL1, BCR, ETV6, and RUNX1* suggest that secondary genetic alterations may further drive disease aggressiveness and therapeutic resistance.

Our review of 11 *KMT2A*-amplified B-ALL cases (two from our cohort and nine published) highlights key clinical trends. Most patients (82%) were older adults (median age 66), with a poor prognosis, as 81.8% succumbed to the disease. The two survivors were pediatric cases, suggesting age may influence prognosis due to differences in disease biology or treatment response. The observed outcome difference between the deceased and remission groups likely reflects an age-associated difference in clinical course, with pediatric cases clustering in the remission group and older adults predominating among fatal cases. However, this observation is presented for exploratory and descriptive purposes only, as formal statistical comparison is limited by marked age imbalance and small sample size, which limit adjustment for confounders. The CD10 negativity (63%) was common, aligning with the aggressive nature of *KMT2A*-rearranged B-ALL. Pre-B ALL accounted for 18% of cases, therapy-related B-ALL for 27%, and B-ALL to T-ALL transformation for 9%, with one case of Ph-like B-ALL further expanding the spectrum of high-risk genetic alterations. Given the poor survival and limited treatment options, novel therapeutic strategies are urgently needed. Standard chemotherapy appears ineffective, as shown by rapid disease progression and high mortality. The co-occurrence of *KMT2A* amplification in this B-ALL entity with *TP53* pathogenic variants or *CRLF2* rearrangement suggests that targeted therapies, including p53 reactivators, JAK inhibitors, and/or epigenetic modulators, should be explored. The potential role of immunotherapy, such as CAR-T cells or bispecific antibodies, also warrants further investigation.

## Data Availability

The original contributions presented in the study are included in the article/**Supplementary Material**. Further inquiries can be directed to the corresponding author.

## References

[1] Castiglioni S Di Fede E Bernardelli C Lettieri A Parodi C Grazioli P . KMT2A: umbrella gene for multiple diseases. Genes (Basel). (2022) 13:514. doi: 10.3390/genes13030514, PMID: 35328068 PMC8949091

[2] Gorecki M Koziol I Kopystecka A Budzynska J Zawitkowska J Lejman M . Updates in KMT2A gene rearrangement in pediatric acute lymphoblastic leukemia. Biomedicines. (2023) 11:746. doi: 10.3390/biomedicines11030821, PMID: 36979800 PMC10045821

[3] Hernandez-Sanchez A Gonzalez T Sobas M Strang E Castellani G Abaigar M . Rearrangements involving 11q23.3/KMT2A in adult AML: mutational landscape and prognostic implications - a HARMONY study. Leukemia. (2024) 38:1929–37. doi: 10.1038/s41375-024-02333-4, PMID: 38965370 PMC11347382

[4] Hess JL . Mechanisms of transformation by MLL. Crit Rev Eukaryot Gene Expr. (2004) 14:235–54. doi: 10.1615/CritRevEukaryotGeneExpr.v14.i4.10, PMID: 15663355

[5] Tang G DiNardo C Zhang L Ravandi F Khoury JD Huh YO . MLL gene amplification in acute myeloid leukemia and myelodysplastic syndromes is associated with characteristic clinicopathological findings and TP53 gene mutation. Hum Pathol. (2015) 46:65–73. doi: 10.1016/j.humpath.2014.09.008, PMID: 25387813

[6] Cuthbert G Thompson K McCullough S Watmore A Dickinson H Telford N . MLL amplification in acute leukaemia: a United Kingdom Cancer Cytogenetics Group (UKCCG) study. Leukemia. (2000) 14:1885–91. doi: 10.1038/sj.leu.2401919, PMID: 11069023

[7] Andersen MK Christiansen DH Kirchhoff M Pedersen-Bjergaard J . Duplication or amplification of chromosome band 11q23, including the unrearranged MLL gene, is a recurrent abnormality in therapy-related MDS and AML, and is closely related to mutation of the TP53 gene and to previous therapy with alkylating agents. Genes Chromosomes Cancer. (2001) 31:33–41. doi: 10.1002/gcc.1115, PMID: 11284033

[8] Wren C Rebeiro P Tegg E . KMT2A amplification in B lymphoblastic leukaemia. Pathology. (2023) 55:738–40. doi: 10.1016/j.pathol.2022.12.356, PMID: 36964033

[9] Wang HY Louis HMS Costello CL Murray SS Dell’Aquila ML . A CD10-negative adult B-lymphoblastic leukaemia with amplification of KMT2A without rearrangement: A case report and review of the English literature. Br J Haematol. (2024) 205:364–7. doi: 10.1111/bjh.19520, PMID: 38735761

[10] Robles AI Jen J Harris CC . Clinical outcomes of TP53 mutations in cancers. Cold Spring Harb Perspect Med. (2016) 6:a026294. doi: 10.1101/cshperspect.a026294, PMID: 27449973 PMC5008065

[11] Rivlin N Brosh R Oren M Rotter V . Mutations in the p53 Tumor Suppressor Gene: Important Milestones at the Various Steps of Tumorigenesis. Genes Cancer. (2011) 2:466–74. doi: 10.1177/1947601911408889, PMID: 21779514 PMC3135636

[12] Jain N Roberts KG Jabbour E Patel K Eterovic AK Chen K . Ph-like acute lymphoblastic leukemia: a high-risk subtype in adults. Blood. (2017) 129:572–81. doi: 10.1182/blood-2016-07-726588, PMID: 27919910 PMC5290985

[13] Alghandour R Sakr DH Shaaban Y . Philadelphia-like acute lymphoblastic leukemia: the journey from molecular background to the role of bone marrow transplant-review article. Ann Hematol. (2023) 102:1287–300. doi: 10.1007/s00277-023-05241-2, PMID: 37129698 PMC10181978

[14] Roberts KG Li Y Payne-Turner D Harvey RC Yang YL Pei D . Targetable kinase-activating lesions in Ph-like acute lymphoblastic leukemia. N Engl J Med. (2014) 371:1005–15. doi: 10.1056/NEJMoa1403088, PMID: 25207766 PMC4191900

[15] Gupta DG Varma N Sreedharanunni S Abdulkadir SA Naseem S Sachdeva MUS . ‘Evaluation of adverse prognostic gene alterations & MRD positivity in BCR::ABL1-like B-lineage acute lymphoblastic leukaemia patients, in a resource-constrained setting. Br J Cancer. (2023) 129:143–52. doi: 10.1038/s41416-023-02294-y, PMID: 37156894 PMC10307811

[16] Gao M Chen Y Lertwilaiwittaya P Hurst ACE Al-Beshri A Carroll AJ . Constitutional mosaic pericentromeric trisomy 8 in a female patient with aplastic anemia. Am J Med Genet A. (2025) 197:e63951. doi: 10.1002/ajmg.a.63951, PMID: 39622782

[17] Harada S Calio A Janowski KM Morlote D Rodriguez Pena MD Canete-Portillo S . Diagnostic utility of one-stop fusion gene panel to detect TFE3/TFEB gene rearrangement and amplification in renal cell carcinomas. Mod Pathol. (2021) 34:2055–63. doi: 10.1038/s41379-021-00858-y, PMID: 34148064

[18] Hanbazazh M Harada S Reddy V Mackinnon AC Harbi D Morlote D . The interpretation of sequence variants in myeloid neoplasms. Am J Clin Pathol. (2021) 156:728–48. doi: 10.1093/ajcp/aqab039, PMID: 34155503

[19] Mater DV Goodman BK Wang E Gaca AM Wechsler DS . MLL duplication in a pediatric patient with B-cell lymphoblastic lymphoma. J Pediatr Hematol Oncol. (2012) 34:e120–3. doi: 10.1097/MPH.0b013e3182273b57, PMID: 22052166

[20] Espinet B Florensa L Salido M Sole F . MLL intrachromosomal amplification in a pre-B acute lymphoblastic leukemia. Haematologica. (2003) 88:EIM03. 12604431

[21] Racke F Cole C Walker A Jones J Heerema NA . Therapy-related pro-B cell acute lymphoblastic leukemia: report of two patients with MLL amplification. Cancer Genet. (2012) 205:653–6. doi: 10.1016/j.cancergen.2012.11.001, PMID: 23238285 PMC4032176

[22] Keung Y-KH Jensen Hu E . Therapy-related acute lymphoblastic leukemia associated with MLL amplification in a patient with metastatic uterine leiomyosarcoma. Proc UCLA Health. (2015) 19.

[23] Takeda R Yokoyama K Fukuyama T Kawamata T Ito M Yusa N . Repeated lineage switches in an elderly case of refractory B-cell acute lymphoblastic leukemia with MLL gene amplification: A case report and literature review. Front Oncol. (2022) 12:799982. doi: 10.3389/fonc.2022.799982, PMID: 35402256 PMC8983914

[24] Chiang YT Chien YC Lin YH Wu HH Lee DF Yu YL . The function of the mutant p53-R175H in cancer. Cancers (Basel). (2021) 13:4088. doi: 10.3390/cancers13164088, PMID: 34439241 PMC8391618

[25] Felix CA Megonigal MD Chervinsky DS Leonard DG Tsuchida N Kakati S . Association of germline p53 mutation with MLL segmental jumping translocation in treatment-related leukemia. Blood. (1998) 91:4451–6. 9616138

[26] Issa GC Aldoss I DiPersio J Cuglievan B Stone R Arellano M . The menin inhibitor revumenib in KMT2A-rearranged or NPM1-mutant leukaemia. Nature. (2023) 615:920–4. doi: 10.1038/s41586-023-05812-3, PMID: 36922593 PMC10060155

[27] Wang ES Issa GC Erba HP Altman JK Montesinos P DeBotton S . Ziftomenib in relapsed or refractory acute myeloid leukaemia (KOMET-001): a multicentre, open-label, multi-cohort, phase 1 trial. Lancet Oncol. (2024) 25:1310–24. doi: 10.1016/S1470-2045(24)00386-3, PMID: 39362248

[28] Böhm JW Sia KCS Jones C Evans K Mariana A Pang I . Combination efficacy of ruxolitinib with standard-of-care drugs in CRLF2-rearranged Ph-like acute lymphoblastic leukemia. Leukemia. (2021) 35:3101–12. doi: 10.1038/s41375-021-01248-8, PMID: 33895784

[29] Kantarjian H Stein A Gokbuget N Fielding AK Schuh AC Ribera JM . Blinatumomab versus chemotherapy for advanced acute lymphoblastic leukemia. N Engl J Med. (2017) 376:836–47. doi: 10.1056/NEJMoa1609783, PMID: 28249141 PMC5881572

[30] Kantarjian HM DeAngelo DJ Stelljes M Martinelli G Liedtke M Stock W . Inotuzumab ozogamicin versus standard therapy for acute lymphoblastic leukemia. N Engl J Med. (2016) 375:740–53. doi: 10.1056/NEJMoa1509277, PMID: 27292104 PMC5594743

[31] Maude SL Laetsch TW Buechner J Rives S Boyer M Bittencourt H . Tisagenlecleucel in children and young adults with B-cell lymphoblastic leukemia. N Engl J Med. (2018) 378:439–48. doi: 10.1056/NEJMoa1709866, PMID: 29385370 PMC5996391

