# Supplementary material for: Case Report: KMT2A amplification in two adult patients with B-cell acute lymphoblastic leukemia
Source: Front Oncol. 2026 Feb 26;16:1656404. doi: 10.3389/fonc.2026.1656404 (PMC12979107; doi:10.3389/fonc.2026.1656404)
Supplement: Supplementary file 1 [file Table1.docx]

**Table S1. Summary of diagnostic tests and clinical outcome of our two patients**

| **Tests/Outcome** | **Patient 1** | **Patient 2** |
| --- | --- | --- |
| **Flow Cytometry** | 54.11% blasts, positive for CD13dim, CD19, CD22dim, CD34, CD38, CD45, CD58, nTdT (B-ALL) | 89.71% blasts, positive for CD10, CD13, CD19, CD22dim, CD34, CD38, CD58, nTdT (B-ALL) |
| **FISH** | *KMT2A*: 6~18 copies in 68.5% of cells | *KMT2A*: 8~11copies in 87.5% of cells; *CRLF2*: rearrangement in 85% of cells; *TP53*: no deletion. |
| **Chromosome Analysis** | Stemline clone with complex karyotype involving chromosomes 3, 5, 10, 11, 14, 16, 18, and 20, in addition to the presence of 1~3 marker chromosomes, and a subclone that represents the inexact doubling product of the stemline clone | Failed; additional material at 11q23 |
| **NGS Analysis** | *TP53*:c.524G>A, p.Arg175His, 91% variant allele frequence | Not done |
| **Outcome** | Induction chemotherapy; died after 2 months due to infections and respiratory failure | Induction chemotherapy; died after 4 days due to multiorgan failure |
